# Advances of Synthesis Methods for Porous Silicon-Based Anode Materials

**DOI:** 10.3389/fchem.2022.889563

**Published:** 2022-04-25

**Authors:** Fan Zhang, Wenqiang Zhu, Tingting Li, Yuan Yuan, Jiang Yin, Jianhong Jiang, Lishan Yang

**Affiliations:** ^1^ Key Laboratory of Chemical Biology and Traditional Chinese Medicine Research (Ministry of Education of China), National and Local Joint Engineering Laboratory for New Petrochemical Materials and Fine Utilization of Resources, Key Laboratory of the Assembly and Application of Organic Functional Molecules of Hunan Province, Hunan Normal University, Changsha, China; ^2^ Hunan Engineering Research Center for Water Treatment Process and Equipment, China Machinery International Engineering Design & Research Institute Co., Ltd., Changsha, China

**Keywords:** silicon anodes, nanomaterials, porous, synthesis, lithium-ion batteries

## Abstract

Silicon (Si)-based anode materials have been the promising candidates to replace commercial graphite, however, there are challenges in the practical applications of Si-based anode materials, including large volume expansion during Li^+^ insertion/deinsertion and low intrinsic conductivity. To address these problems existed for applications, nanostructured silicon materials, especially Si-based materials with three-dimensional (3D) porous structures have received extensive attention due to their unique advantages in accommodating volume expansion, transportation of lithium-ions, and convenient processing. In this review, we mainly summarize different synthesis methods of porous Si-based materials, including template-etching methods and self-assembly methods. Analysis of the strengths and shortages of the different methods is also provided. The morphology evolution and electrochemical effects of the porous structures on Si-based anodes of different methods are highlighted.

## 1 Introduction

Portable electronic devices and electric vehicles (EV) are gradually integrated into people’s daily life, thus improving the energy density of batteries is critical. (Yu et al., 2014; Choi and Aurbach, 2016) Nowadays, commercial graphite anodes couldn’t meet people’ growing needs. (Tarascon and Armand, 2001; Choi et al., 2012)Alloy-type anode materials have been widely studied due to their high theoretical specific capacity. (Wu et al., 2014) Among them, Si-based anode materials possess the highest theoretical capacity (4,200 mA h g^−1^, corresponding to fully lithiated Li_22_Si_5_ alloys) to replace graphite as next-generation superior anode materials. (Hui and Yi, 2012; McDowell et al., 2013; Luo et al., 2017; Zhou et al., 2022) Many researches have been reported recently about Si-based anodes. (Lai, 1976; Chae et al., 2017; Feng et al., 2018) Actually, the huge volume expansion (∼420%) of Si-based anode materials during charging/discharging processes severely limits their practical applications, which leads to materials pulverization, electrode failure and unstable solid electrolyte interphase (SEI). (Kim et al., 2014; Rahman et al., 2016; Jin et al., 2017) Besides, the low intrinsic conductivity of Si also limits its applications to some extent.

**FIGURE 1:**
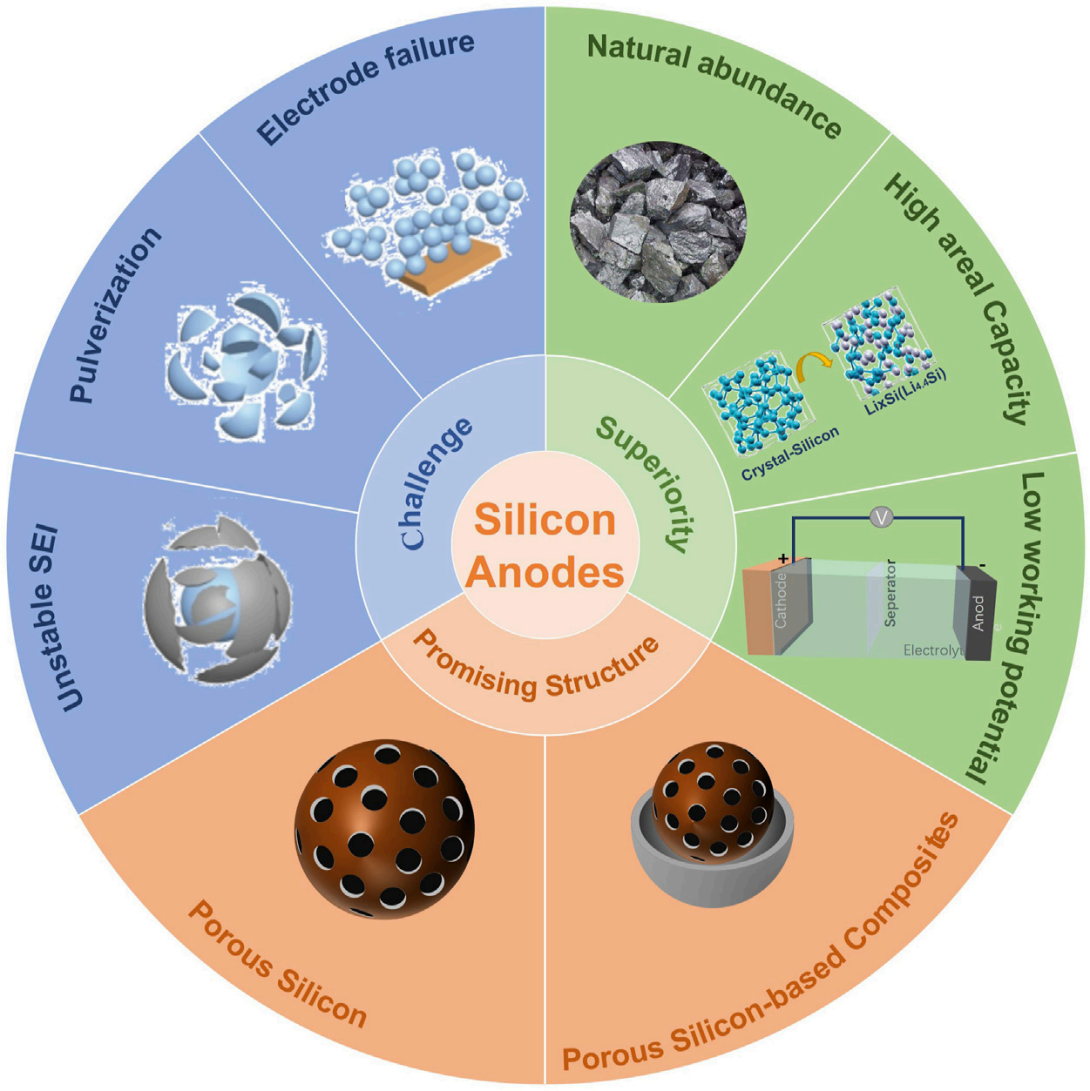
An overview of silicon anodes: “Challenge,” “Superiority” and “Promising Structure.” The inserted graphics are adapted with permission from (Johari et al., 2011; Jin et al., 2017).

To address the issues associated with Si, especially volume expansion, massive efforts have been made, including: avoiding materials pulverization via the design of silicon nanostructures, (An et al., 2020; Qi et al., 2020; Sun et al., 2022a; Sun et al., 2022b; Li et al., 2022) improving cycling stability through SiO/SiO_x_-based anode materials, (Wang et al., 2020a; Tian et al., 2022) and increasing electronic/ionic conductivities through utilizing advanced electrolyte additives and novel binders. (Huang et al., 2019; Zhao et al., 2021; Zhou et al., 2021; Zhu et al., 2021) The 3D porous Si-based materials have enough internal voids to accommodate volume expansion due to the existence of their large pores, so that their structures can maintain their integrity during the processes of lithiation/delithiation, avoiding pulverization of silicon-based materials. (Ashuri et al., 2016; Dou et al., 2019) In addition, the large specific surface area of 3D porous Si-based materials is beneficial to the transport of Li^+^, thus improving the rate performance of Si-based materials. (Ge et al., 2013; Manj et al., 2018) Based on this, 3D porous Si-based anodes have received extensive attention, which is shown in Figure 1.

**FIGURE 2:**
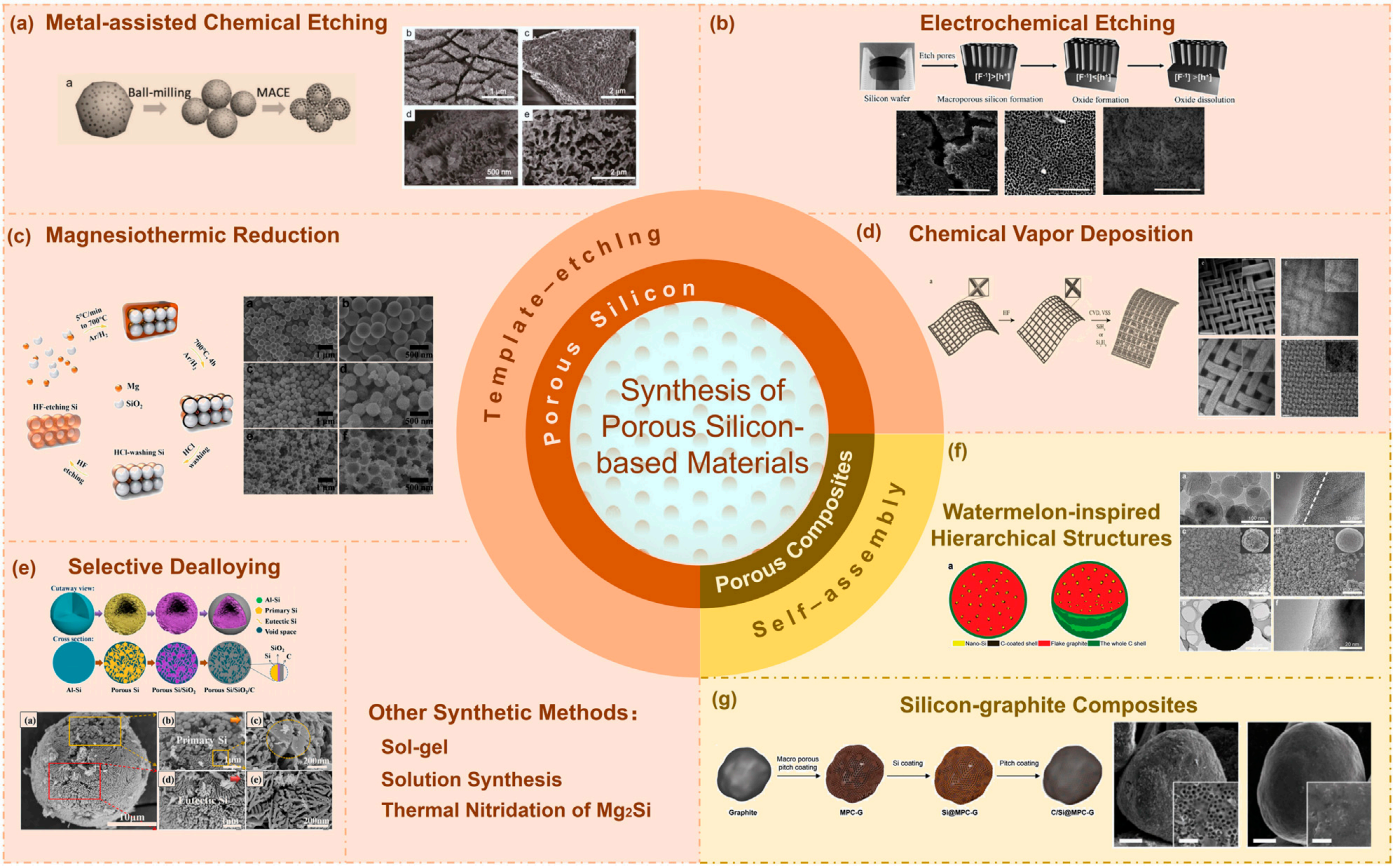
The various synthetic routes and structural characteristics of different methods for porous silicon-based materials. The inserted graphics are adapted with permission from (Thakur et al., 2012; Jin et al., 2015; Xu et al., 2017; Zuo et al., 2017; Harpak et al., 2019; Wang et al., 2019; Son et al., 2020).

In this review, we will summarize different synthesis methods of porous Si-based anode materials, and compare the differences in structures and electrochemical performance between different synthesis methods. The various synthetic routes and structural characteristics of different methods are shown in Figure 2, and the comparison of electrochemical performance between porous Si-based anode materials prepared by different methods is shown in Supplementary Table S1.

## 2 Synthesis of Porous Silicon-Based Materials

Porous silicon has been applied in the field of chemical sensors, solar energy, and LIBs since 1990s. The 3D structures possess their unique advantages, which can relieve the volume stress of Si-based materials.

Porous silicon can be divided into nano-sized structures and micron-sized structures equipped with nano characteristics. Nano-sized silicon materials with large specific surface area can relieve the volume expansion of silicon, thus avoiding cracking of materials and enhancing the cycling performance. On the basis of retaining the advantages of nanoscale, nano-sized porous silicon materials can utilize their porous structures to better accommodate the volume expansion, thereby further improving their mechanical stability. In recent years, various methods of synthesizing nano-sized porous silicon materials have been reported successively, including various etching methods and template-assisted methods. Despite nano-sized porous structures have unique advantages in accommodating the volume expansion, excessive specific surface area and side reactions result in low Columbia efficiency, which limits their practical applications. In addition, the low tap density also needs to be addressed. Micro-sized porous Si-based materials with nano characteristics eliminate the shortages of nano-sized materials while retaining the benefits of them. They have bicontinuous structures of silicon crystals and pores, achieving superior transport of ions and electrons at the micro-sized comparable to those achieved at the nano-sized, and buffering the volume change of silicon crystals. There are two categories to design micro-sized porous Si-based materials. One is the micro-sized porous silicon with nano characteristics, and selective dealloying is a relatively mature method for synthesizing such materials. Another category is the incorporation of nano-silicon crystals onto the micro-nano hybrid porous structures, such as graphite and amorphous carbon.

The above porous structures all have the characteristics of electric double layer capacitance and show good electrochemical performance. In different synthesis methods, structural parameters such as pore size, particle size, and specific surface area can be regulated by changing the synthesis conditions. In the following sections, we will introduce the principles, characteristics and corresponding electrochemical properties of different synthesis methods, summarize the research progress of porous Si-based materials in recent years, put forward the current issues and look forward to their future practical applications.

### 2.1 Synthesis of Porous Silicon

#### 2.1.1 Metal-Assisted Chemical Etching

Metal-assisted chemical etching (MACE) is a typical top-down synthesis method, which utilizes bulk silicon (such as doped silicon wafers as raw materials), and porous Si-based materials are obtained via the galvanic displacement between metal particles such as Ag and Si in HF solution. (Bai et al., 2012) The general MACE process assisted by the introduction of silver particles via adding AgNO_3_ solution to the solution, was described by the following two simultaneous electrochemical reactions:

$$4\text{A}{\text{g}}^{+}+4{\text{e}}^{-}\to 4\text{A}\text{g}$$

$$\text{S}\text{i}+6{\text{F}}^{-}\to {\left[\text{S}\text{i}{\text{F}}_{6}\right]}^{2-}+4{\text{e}}^{-}$$

As for silicon wafer raw materials, a high degree of n-/ p-doping can provide defect sites on the surface of the materials to facilitate the formation of porous structures. Ge et al. synthesized porous silicon nanowires by MACE of boron-doped silicon wafers. (Ge et al., 2012) The experiments indicated that the doping was a necessary condition for the formation of porous structures. If the silicon wafer were not doped, the obtained silicon would be solid silicon nanowires rather than porous structures. The obtained anode materials with porous silicon nanowire, which possessing with both 8 nm pore size and wall thickness, showed good long-term cycling performance as well as a reversible capacities of 1,100 mA h g^−1^ at the rate of 4.5 C after 250 cycles. Meanwhile, they set up a mathematical model to simulate the effect of porosity and pore size on the structural stability, and concluded that high porosity and large pore size could help to stabilize its structures, which is consistent with the experimental results.

Compared with silicon wafers, metallurgical grade silicon (MG-Si) is an attractive material option as a low-grade silicon source and has been extensively studied in recent years due to its cheap price (∼1,000 $/ton) and natural abundance. Jin et al. reported a scalable process involving ball milling and modified MACE. (Jin et al., 2015) Different from traditional MACE, they adjusted the dispersion and hydrophobicity of silicon powder via adding ethanol into the etching solution to obtain porous nanostructures with different morphologies. At the same time, the etching process was accelerated by adding H_2_O_2_ solution, and silicon particles with micro-pores were obtained. The obtained materials with micro/nano-sized structures exhibited excellent electrochemical performance (capacity retention of 80% after 100 cycles) due to their micro-sized porous structures.

#### 2.1.2 Electrochemical Etching

The etching-type methods for preparing porous silicon also include stain etching and electrochemical etching. Stain etching is a simple and scalable method that is widely used in the field of solar energy, (Dimova-Malinovska et al., 1997) but its practical applications in LIBs is rarely reported, because its limited control over reaction parameters results in an inability to selectively control the morphologies. Electrochemical etching is another etching-type method for preparing porous Si-based materials, and silicon wafers are also generally employed as raw materials. Different from MACE’s method of etching silicon wafers with the assistance of noble metal particles, electrochemical etching is to etch silicon wafers through HF solution at a constant current density, and the pore size, pores and other structural parameters of the materials can be varied by changing the current density. Thakur et al. reported a simple method to prepare thin films of macro-porous silicon, which can achieve a discharge capacity of 1,260 mA h g^−1^ when combined with pyrolytic polyacrylonitrile. (Thakur et al., 2012) However, compared with MACE, this method has drawbacks in controlling the material morphologies and scaling up production, which couldn’t be widely utilized in practical applications.

#### 2.1.3 Magnesiothermic Reduction

Magnesiothermic reduction method possesses the potential to expand the production of porous silicon materials because of its ability to maintain the original structures of the template. Unlike conventional carbothermic reduction, the reaction temperature of this method is relatively low (500–950°C), which is much lower than the melting point of silicon (1,414°C). (Entwistle et al., 2018) The reactions that take place in the process are as follows:

$$\text{S}\text{i}{\text{O}}_{2}+2\text{M}\text{g}\to 2\text{M}\text{g}\text{O}+\text{S}\text{i}$$

$$\text{S}\text{i}+2\text{M}\text{g}\to \text{M}{\text{g}}_{2}\text{S}\text{i}$$

Magnesium oxide and silicon are formed by a simple reduction reaction of silicon dioxide with magnesium, and the silica near the magnesium source will be reduced to form Mg_2_Si by-product during the reaction. All by-products including excess magnesium, unreacted silicon dioxide, magnesium oxide and Mg_2_Si were removed after etching by HCl and HF solution, respectively, resulting in porous silicon-based materials with specific template structures.

The reaction utilizes silica as template and starting material. It is known that silica is abundant in nature. (Zhang et al., 2016)Among various biological sources, rice husks are considered as potential silicon sources for large-scale production of porous silicon due to their low cost and recyclability. Jung et al. converted silica in rice husk into silicon with excellent electrochemical performance as anode materials, indicating that rice husk is a potential silicon source for large-scale applications. (Jung et al., 2013) Jiao et al. prepared porous Si-based materials via utilizing rice husks as silicon precursors by the same process, and uniformly inserted them into graphene by combining a condensation reaction and subsequent thermal treatment. (Jiao et al., 2016) It was found that the composites exhibited good cycling stability (830 mA h g^−1^ at 1 A g^−1^ after 200 cycles) and rate performance. The performance improvements stem from the buffering effect of graphene and the porous structures on volume expansion.

Although the above methods have the potential to expand production due to their low cost, however, silica materials derived from biological resources generally contain carbon, and silicon carbide may be formed in places with high local temperature during the magnesium reduction processes, leading to applications restricted. Besides, some impurities are generally present in the natural silica precursors, and additional processes are required to remove the impurities, resulting in increased cost. Artificial silica has also received extensive attention due to its high purity and controllable size. Nowadays, the most widely used artificial silica is from tetraethylorthosilicate (TEOS), because this method can obtain nanoparticles (about 30–60 nm) with excellent monodispersity. (Xiao et al., 2015) Zuo et al. synthesized nano-sized silica spheres via using TEOS as the silicon precursor, and 3D hierarchical macroscopic/mesoporous silicon powders were synthesized through the magnesiothermic reduction process. (Zuo et al., 2017) A reversible capacity of 959 mA h g^−1^ was retained at 0.2 A g^−1^ after 300 cycles. The macropores accommodate the drastic volume expansion of the silicon under lithiation, and the mesopores facilitate Li^+^ transport due to their larger specific surface area. Even though the production process of artificial silica is complicated, the method still possesses certain research value owing to its high purity and structural design characteristics.

#### 2.1.4 Chemical Vapor Deposition

The preparation of porous silicon by CVD involves etching templates, also classified as a template-etching method. Silica, ZnO nanowire arrays, porous Ni materials, etc. can be employed as porous templates to realize the preparation of porous silicon. Tesfaye et al. fabricated silicon nanotubes with thin porous sidewalls by utilizing ZnO nanowires as sacrificial templates, maintaining a reversible capacity of 800 mA h g^−1^ at C/10 after 180 cycles. (Tesfaye et al., 2015) Harpak et al. prepared 3D, sponge-like silicon nanostructure network on nano-porous stainless steel surface by CVD. (Harpak et al., 2019) These novel anodes exhibited stable cycle performance with over 50% capacity retention after 500 cycles and high Coulombic efficiency (over 99.5%).

The uniform surface coating and growth of the materials on the substrate can be achieved by CVD, and the prepared porous Si-based composite materials possess good cycle life and rate performance, but silane as a general silicon source is expensive, toxic, and explosive, which limits its applications along with safety hazards. Therefore, it is urgent to find safer and cheaper alternative silicon sources.

#### 2.1.5 Selective Dealloying

Although nano-sized silicon materials possess unique advantages in relieving volume stress, the inherent shortcomings such as unstable SEI films, low tap density and excessive side reactions have severely limited their practical applications. Therefore, the porous Si-based materials with both micro-nano structures are of great practical significance. They retain advantages of nanostructures while increasing their tap density through microstructures. Recently, selective dealloying is considered as a promising method for manufacturing micro-porous silicon. (Luo et al., 2020; Zhou et al., 2020) This method employs cheap silicon-metal alloys (such as Si-Al alloys etc.) as raw materials, and porous silicon materials are obtained by removing metal elements in the alloys. Wang et al. prepared the double-layer constrained micron-sized porous Si/SiO_2_/C composites by dealloying, the obtained coral-like porous silicon structures exhibited good cycle stability with the capacity of 933.4 mA h g^−1^ after 100 cycles. (Wang et al., 2019) Although this method requires a large amount of acid for etching, the silicon-alloy precursors of this method are easy to achieve large-scale manufacture, which is promising for further applications.

#### 2.1.6 Other Synthetic Methods

In addition to the above methods, other methods such as molten salt, solution synthesis, sol-gel, preparation of porous Si-based materials using Mg_2_Si as silicon precursor, etc., have also been reported in recent years. The metals magnesium or aluminum have been demonstrated to reduce silica into silicon. However, excessive reaction temperature of magnesiothermic reduction limits its applications (500–950°C). Recently, molten salt systems have been considered as a promising reaction media to reduce silica at a lower temperature. (Liu et al., 2012; Mishra et al., 2018) Lin et al. designed a low temperature molten salt system for metallothermic reduction of silica and silicates. The AlCl_3_ in this system could not only provide liquid environment, but also participate in the reaction. This anode showed a reversible specific capacity of 870 mA h g^−1^ at 3 A g^−1^ after 1,000 cycles (Lin et al., 2015). Gao et al. prepared a silicon hollow sphere by reduction of a carbon-coated silica sphere at 300°C with metal Al powder in a molten salt. After being prelithiated, it retains 80% of the initial capacity after 1,100 cycles at 8 A g^−1^ (Gao et al., 2018). Molten salt system enables controllable preparation of porous silicon at lower temperatures. It is noticed that AlCl_3_ is very sensitive to water, the reactions need conduct in stainless steel autoclave to isolate air. The method of low temperature solution synthesis of silicon has been widely concerned because of its mild conditions. Nanostructured Si such as nanoparticles, nanosheets and porous materials have been prepared through reduction reaction of silane and silicon halide. (Wang et al., 2020b; Wang et al., 2020c) Sun et al. prepared mesoporous silicon in high yields by a simple solution chemistry method. (Sun et al., 2017) After crystallization and carbon coating, the crystalline mesoporous Si/C nanocomposites exhibited high reversible capacity with 847 mA h g^−1^ at 2 A g^−1^ after 320 cycles.

Besides selective dealloying, there are other methods to synthesis micro-sized porous silicon. An et al. fabricated ant-nest-like microscale porous Si with nano-porous from Mg_2_Si. The porous silicon materials exhibited good cycling performance after carbon coating (a reversible capacity of 1,271 mA h g^−1^ after 1,000 cycles). (An et al., 2018) Liu et al. prepared interpenetrating 3D porous silicon by preparing a novel silica composite gel and then performing a modified magnesium thermal reduction process. (Liu et al., 2018) This method retained the bicontinuous structures of gel, and the cycling stability and rate performance were improved (0.98 mA h cm^−2^ after 200 cycles). Although the above methods can controllably obtain the micro-sized porous silicon with nano characteristics, the inevitable shortages in their synthesis process limit their applications. The use of high-cost commercial Mg_2_Si during the reaction process increases production costs of porous silicon. Although sol-gel method provides an unconventional solution to the capacity fading of Si-based anodes, complex multi-step synthesis increases the production cost and production period, which is not conducive to the expansion of large-scale manufacture, and remains to be further improved.

### 2.2 Synthesis of Porous Silicon-Based Composites

Besides above-mentioned porous silicon with nanostructures and both micro-nano structures, there are also many reports on the preparation of silicon/carbon composites by self-assembly recently. A method widely favored by the industry is the incorporation of nano-silicon crystals onto the micro-nano hybrid porous structure of graphite. (Yoshio et al., 2006; Datta and Kumta, 2007) Graphite is the current commercial anode material with good cycle stability, while Si-based materials need to achieve high capacity while retaining the advantages of graphite. Most silicon/graphite composites are prepared by mechanical mixing, which inevitably causes structural damage to the graphite, resulting in rapid electrode failure. (Lu et al., 2018) Therefore, the content of silicon in these micro-sized Si/C composites is low, so that the small volume expansion of the inner nano-silicon particles hardly damages the integrity structure of the outer graphite, thus achieving a stable SEI layer and high Coulombic efficiency (CE). Ko et al. synthesized silicon-nanolayer-embedded graphite/carbon hybrids (SGC) by CVD. (Ko et al., 2016) The composites electrode achieves high volumetric energy density (1,043 Wh L^−1^) and excellent initial Coulombic efficiency (ICE, 92%).

Another promising approach is to integrate nano-silicon with different carbon frameworks to form a micro-silicon-carbon composites structure. Technologies such as CVD and spray drying are widely employed to prepare such materials. (Xu et al., 2018) In this way, the micro-scale materials with uniformly distributed nano-silicon help to form a stable SEI layer on the surface due to their small specific surface area. Meanwhile, the voids inside the hierarchical micro-materials help alleviate the volume expansion of silicon. Xu et al. prepared watermelon-inspired Si/C microspheres by CVD and spray drying, and the hierarchical Si/C anode with high tap density showed excellent cycling performance (capacity retention of 80% after 250 cycles) and rate capability. (Xu et al., 2017) Similar design of composites structures has a guiding role in improving the performance of Si-based materials, which is beneficial to its practical applications.

## 3 Conclusion and Perspective

Silicon is considered as promising next-generation anode materials for high-energy-density LIBs due to its much higher theoretical specific capacity than commercial graphitic anode materials, but a series of issues caused by volume expansion hinder the commercialization of Si-based anode materials. The porous Si-based anode materials with nano-characteristics have enough internal voids to accommodate the volume expansion of silicon, and their larger specific surface is conducive to the transport of ions and electrons, thus enhancing the electrochemical performance. In this review, we focus on the different synthesis methods of porous Si-based materials, and compare their structural and performance differences. 3D porous silicon exhibits more stable cycling performance than 0, 1 and 2D nanomaterials because of its unique structures. Their unique porous structures help improve cycling stability, and their excellent ionic-electronic conductivity enables them to exhibit better rate performance. The cycle performance of nano-sized porous silicon needs to be improved due to the agglomeration of nanomaterials and excessive specific surface area. Micro-sized porous Si-based materials with nano characteristics exhibit better cycling stability and rate performance, promising for commercial applications.

Various methods have been developed to design porous silicon-based materials, all of which exhibit high capacity and long-cycle stability. Micro-sized porous Si-based materials with nano characteristics have certain applications prospects, but how to further simplify their preparation process and optimize the cost still needs further exploration. In addition, the match of porous Si-based materials and advanced electrolyte additives as well as novel binders remains to be further explored. More advanced characterization technologies are also applied to analysis the electrochemical processes, such as cryo-EM, SIMS, *In-situ* characterization techniques, etc.
