# Supplementary material for: Advances of Synthesis Methods for Porous Silicon-Based Anode Materials
Source: Front Chem. 2022 Apr 25;10:889563. doi: 10.3389/fchem.2022.889563 (PMC9081600; doi:10.3389/fchem.2022.889563)
Supplement: Supplementary file 1 [file Table1.docx]

**Supporting information**

**Advances of Synthesis Methods for Porous Silicon-Based Anode Materials**

**Fan Zhang ^1^, Wenqiang Zhu ^1^, Tingting Li ^1^, Yuan Yuan ^1^, Jiang Yin ^1,*^, Jianhong Jiang ^2^, Lishan Yang ^1,*^**

^1^ Key Laboratory of Chemical Biology & Traditional Chinese Medicine Research (Ministry of Education of China), National and Local Joint Engineering Laboratory for New Petrochemical Materials and Fine Utilization of Resources, Key Laboratory of the Assembly and Application of Organic Functional Molecules of Hunan Province, Hunan Normal University, Changsha, Hunan 410081, P.R. China

^2^ Hunan Engineering Research Center for Water Treatment Process & Equipment, China Machinery International Engineering Design & Research Institute Co., Ltd., Changsha 410007, China.

*** Correspondence:**

yinjiang33@126.com (Jiang Yin)

lsyang.chemistry@gmail.com (Lishan Yang)

Tel./Fax.: +86 731 88872531

**Table S1.** Summary of synthesis methods and corresponding electrochemical properties of porous silicon anodes materials in LIBs.

| Category | Raw materials | Synthesis methods | Anode active materials | Initial Capacity/  (mA h g^−1^) | Initial CE | Capacity retention | *Ref.* |
| --- | --- | --- | --- | --- | --- | --- | --- |
| Non-template | Metallurgical-grade Si | Ball-milling, metal-assisted chemical  etching | Porous Si | 1250 | ~70% | 1000 mA h g^−1^ after 100 cycles at 0.42 A g^−1^ | [1] |
|  | Silane gas | PECVD, metal-assisted chemical  etching , PECVD | Network of Interconnected porous Si nanowires coated with thin carbon | 3188 | 93.8% | 1275 mA h g^−1^ after 500 cycles at 25 A g^−1^ | [2] |
|  | Boron-doped Si wafers | Metal-assisted chemical  etching | Porous boron-doped Si Nanowires | 3354 | 90.6% | 1960 mA h g^−1^ after 200 cycles at 2 A g^−1^ | [3] |
|  | Metallurgical Si | Metal-assisted chemical  etching | Porous Si nanowires stabilized by a surface oxide layer (Si/SiO_x_) | 4513 | 42% | 1802 mA h g^−1^ after 100 cycles at 0.8 A g^−1^ | [4] |
|  | Bulk Si | Silver (Ag) deposition, metal-assisted chemical etching, thermal decomposition method of acetylene gas | Carbon-coated porous Si | 2530 | 94.4% | 2201 mA h g^−1^ after 50 cycles at 0.84 A g^−1^ | [5] |
|  | Metallurgical-grade Si powder | Rochow reaction, acid washing, carbonization, HF etching | Porous Si/C+C-900-HF | 1241.7 | 69% | 731.2 mA h g^−1^ after 100 cycles at 0.05 A g^−1^ | [6] |
|  | SiCl_4_ | Electrodeposition | Amorphous nanosized Si with hierarchically porous | 1200 | ~84% | 600 mA h g^−1^ after 1000 cycles at 2 A g^−1^ | [7] |
|  | Boron-doped Si wafers | Electrochemical  etching, rapid Thermal Annealing | porous Si films | 1302 | 58.8% | 616.3 mA h g^−1^ after 100 cycles at 0.716 A g^−1^ | [8] |
|  | Template |  |  |  |  |  |  |
| Template Assisted | Hydrogen gas bubble | two-step electrodeposition process using a hydrogen gas  bubble template | 3D porous Si/  copper films | 5548 | 50.9% | 1628 mA h g^−1^ after 50 cycles at 0.84 A g^−1^ | [9] |
|  | ZnO nanowire | CVD | Porous Si nanotube arrays | 4192 | 63% | 1670 mA h g^−1^ after 30 cycles at 0.21 A g^−1^ | [10] |
|  | Porous Ni | CVD | 3D porous Si  particles | 2680 | ~78% | 1650 mA h g^−1^ after 120 cycles at 4.2 A g^−1^ | [11] |
|  | Commercial Mg_2_Si alloy | Vacuum distillation | Nanoporous Si | 3412 | 85% | 1180 mA h g^−1^ after 400 cycles at 1 A g^−1^ | [12] |
|  | Mg and  metallurgical Si | Alloying the bulk metallurgical Si and Mg, nitrided under N_2_,  acid etching, carbon coating | Ant-nest-like bulk porous  Si@C | 2843 | 80.3% | 1271 mA h g^−1^ after 1000 cycles at 2.1 A g^−1^ | [13] |
|  | Commercially available Mg_2_Si particles | Ball-ball milled, annealed under air atmosphere, acid etching | Porous Si  microparticle | 3191 | 86.1% | 1375 mA h g^−1^ after 100 cycles at 2.1 A g^−1^ | [14] |
|  | micellar bicontinuous microemulsion | TEOS hydrolysis, condensation reaction, magnesiothermic reduction, carbon coating | Carbon-coated Si nanoparticles | ~2870 | ~51.6% | 1036 mA h g^−1^ after 100 cycles at 0.5 A g^−1^ | [15] |
|  | SSZ-13 (the  content of silica is ~90 wt%) | Magnesiothermic reduction, carbon coating by CVD | Carbon-coated CM-Si | 2309 | 74.7% | 1338 mA h g^−1^ after 200 cycles at 2 A g^−1^ | [16] |
|  | Nanosized SiO_2_ powder | Ball-milling, magnesiothermic reduction | Porous coral-like Si particles | 3088 | 79.4% | 1956 mA h g^−1^ after 100 cycles at 0.72 A g^−1^ | [17] |
|  | Al−Si alloy particles | Acid etching, heating at 100 °C under the atmosphere, carbon coating | Micron-sized porous  Si/SiO_2_/C composites | 1814.1 | 53.5% | 933.3 mA h g^−1^ after 100 cycles at 0.1 A g^−1^ | [18] |
|  | Cgel,  composed of hydrolysate of TEOS and copper salts | Magnesiothermic reduction | Porous Si | 2305 | 74.5% | 1195.2 mA h g^−1^ after 200 cycles at 0.2 A g^−1^ | [19] |

## REFERENCES

[1] Y. Jin, S. Zhang, B. Zhu, Y. Tan, X. Hu, L. Zong, J. Zhu, Simultaneous purification and perforation of low-grade Si sources for lithium-ion battery anode, Nano Lett., 15 (2015) 7742-7747.

[2] B. Wang, J. Ryu, S. Choi, X. Zhang, D. Pribat, X. Li, L. Zhi, S. Park, R.S. Ruoff, Ultrafast-charging silicon-based coral-like network anodes for lithium-ion batteries with high energy and power densities, ACS Nano, 13 (2019) 2307-2315.

[3] M. Ge, J. Rong, X. Fang, C. Zhou, Porous doped silicon nanowires for lithium ion battery anode with long cycle life, Nano Lett., 12 (2012) 2318-2323.

[4] Y. Chen, L. Liu, J. Xiong, T. Yang, Y. Qin, C. Yan, Porous Si nanowires from cheap metallurgical silicon stabilized by a surface oxide layer for lithium ion batteries, Adv. Funct. Mater., 25 (2015) 6701-6709.

[5] B.M. Bang, J.I. Lee, H. Kim, J. Cho, S. Park, High-performance macroporous bulk silicon anodes synthesized by template-free chemical etching, Adv. Energy Mater., 2 (2012) 878-883.

[6] Z. Zhang, Y. Wang, W. Ren, Q. Tan, Y. Chen, H. Li, Z. Zhong, F. Su, Scalable synthesis of interconnected porous silicon/carbon composites by the rochow reaction as high-performance anodes of lithium ion batteries, Angew. Chem., 126 (2014) 5265-5269.

[7] W. Li, X. Guo, Y. Lu, L. Wang, A. Fan, M. Sui, H. Yu, Amorphous nanosized silicon with hierarchically porous structure for high-performance lithium ion batteries, Energy Storage Mater., 7 (2017) 203-208.

[8] A. Roland, A. Dupuy, D. Machon, F. Cunin, N. Louvain, B. Fraisse, A. Boucherif, L. Monconduit, In-depth study of annealed porous silicon: understand the morphological properties effect on negative LiB electrode performance, Electrochim. Acta, 323 (2019) 134758.

[9] J. Suk, D.W. Kim, Y. Kang, Electrodeposited 3D porous silicon/copper films with excellent stability and high rate performance for lithium-ion batteries, J. Mater. Chem. A, 2 (2014) 2478-2481.

[10] A.T. Tesfaye, R. Gonzalez, J.L. Coffer, T. Djenizian, Porous silicon nanotube arrays as anode material for Li-ion batteries, ACS Appl. Mater. Inter., 7 (2015) 20495-20498.

[11] S.R. Gowda, V. Pushparaj, S. Herle, G. Girishkumar, J.G. Gordon, H. Gullapalli, X. Zhan, P.M. Ajayan, A.L.M. Reddy, Three-dimensionally engineered porous silicon electrodes for Li ion batteries, Nano Lett., 12 (2012) 6060-6065.

[12] Y. An, H. Fei, G. Zeng, L. Ci, S. Xiong, J. Feng, Y. Qian, Green, scalable, and controllable fabrication of nanoporous silicon from commercial alloy precursors for high-energy lithium-ion batteries, ACS Nano, 12 (2018) 4993-5002.

[13] W. An, B. Gao, S. Mei, B. Xiang, J. Fu, L. Wang, Q. Zhang, P.K. Chu, K. Huo, Scalable synthesis of ant-nest-like bulk porous silicon for high-performance lithium-ion battery anodes, Nat. Commun., 10 (2019) 1-11.

[14] J. Wang, W. Huang, Y.S. Kim, Y.K. Jeong, S.C. Kim, J. Heo, H.K. Lee, B. Liu, J. Nah, Y. Cui, Scalable synthesis of nanoporous silicon microparticles for highly cyclable lithium-ion batteries, Nano Res., 13 (2020) 1558-1563.

[15] B. Kim, J. Ahn, Y. Oh, J. Tan, D. Lee, J.-K. Lee, J. Moon, Highly porous carbon-coated silicon nanoparticles with canyon-like surfaces as a high-performance anode material for Li-ion batteries, J. Mater. Chem. A, 6 (2018) 3028-3037.

[16] B. Wang, W. Li, T. Wu, J. Guo, Z. Wen, Self-template construction of mesoporous silicon submicrocube anode for advanced lithium ion batteries, Energy Storage Mater., 15 (2018) 139-147.

[17] D.T. Ngo, H.T. Le, X.-M. Pham, J.-W. Jung, N.H. Vu, J.G. Fisher, W.-B. Im, I.-D. Kim, C.-J. Park, Highly porous coral-like silicon particles synthesized by an ultra-simple thermal-reduction method, J. Mater. Chem. A, 6 (2018) 2834-2846.

[18] K. Wang, Y. Tan, P. Li, B. Xue, J. Sun, Facile synthesis of double-layer-constrained micron-sized porous Si/SiO_2_/C composites for lithium-ion battery anodes, ACS Appl. Mater. Inter., 11 (2019) 37732-37740.

[19] Y.X. Liu, L.J. Qin, F. Liu, Y.M. Fan, J.J. Ruan, S.J. Zhang, Interpenetrated 3D porous silicon as high stable anode material for Li-Ion battery, J. Power Sources, 406 (2018) 167-175.
